# Patients reported outcome post-cochlear implantation: how severe is their dizziness?

**DOI:** 10.1186/s40463-014-0049-z

**Published:** 2014-12-10

**Authors:** Faisal Zawawi, Faisal Alobaid, Tony Leroux, Anthony G Zeitouni

**Affiliations:** Department of Otolaryngology, Head and Neck Surgery, McGill University, 687 Pine Ave West, Montreal, QC Canada; Department Otolaryngology, Head and Neck Surgery, King Abdulaziz University, Jeddah, Saudi Arabia; Department of Audiology, Université de Montréal, Montréal, QC Canada

**Keywords:** Cochlear implants, Dizziness, DHI, Vertigo

## Abstract

**Objectives:**

The reported prevalence of vestibular dysfunction after cochlear implantation (CI) is varies between different scientific papers. The aim of this study is to determine the reported post-implantation outcome in terms of dizziness, and to measure its impact on quality of life using the *Dizziness handicap inventory* (DHI).

**Methods:**

This was a prospective questionnaire based study of postoperative cochlear implant patients. The questionnaire assessed the type and onset of dizziness in addition to the DHI.

**Results:**

122 patients were recruited in this study, which is the largest sample size in the literature reported so far. Dizziness was evident in 45.9% of the population post-CI and in 27% pre-CI. The commonest subtype of the dizziness was unsteadiness followed by lightheadedness. The dizziness was mild in most of the patients.

**Conclusion:**

Although mild, dizziness is a common complaint post-cochlear implantation. An understanding of symptoms helps counsel patients preoperatively.

## Introduction

Many studies have examined the implications on the cochlea of cochlear implantation; however, limited work has been done to analyze the effects on the vestibular system.

Cochlear implantation (CI) is considered the pinnacle of advancement in auditory science of the past few decades. It is estimated that more than 320,000 patients have been implanted. It is currently used as a treatment modality for sensorineural hearing loss (SNHL) [1]. Initially, CI was used on deaf patients [2], but now, CI indications have expanded and bilateral implantation is a recognized modality [2]. There is now emphasis on minimizing trauma to the inner ear and maximizing residual inner ear function.

It has been reported that the incidence of postoperative vestibular dysfunction ranges from 20-70% [3]. Preoperative labyrinthine status and concurrent inner ear diseases such as Meniere’s clearly play some role. This is thought to be the main contributing factor [4]. Other factors described in the literature include vision, proprioception, age and the state of the muscular and cardiovascular systems [4].

Procedure related causes can also lead to vestibular dysfunction. Trauma from inserting the electrode array, cochleostomy induced serous labyrinthitis, perilymph loss intraoperatively, endolymphatic hydrops, and stimulation from the electrodes, are all reported plausible causes for postoperative vestibular dysfunction [3,5–7].

There are several studies focusing on the vestibular complications of cochlear implantation but concluding different results. Hence, further work is still needed that analyzes vestibular function and assesses the effect of implanting the cochlea [8–10].

The aim of this study is to determine patient reported outcome and quality of life post-CI in terms of dizziness as well, as to assess the prevalence of vestibular symptoms pre- and post-CI.

## Methods

### The study was approved by the institutional review board and informed consent from study subjects was obtained

This study is a questionnaire-based review of patients recruited from Institute Raymond-Dewar—a rehabilitation center in Montreal that specializes in hearing and communication.

The population consisted of postoperative CI patients who had profound sensorineural hearing loss prior to the implantation. These patients were recruited and provided French and English versions of the questionnaire by mail to all the adult cochlear implant recipients followed at the IRD. Patients answered the questionnaire once. None had a bilateral implantation. The questionnaire included the Dizziness Handicap Inventory (DHI) which was used to assess the impact of vestibular dysfunction on the patients’ daily activities. and questions asking the type of dizziness and its onset [11,12]. The classification and definitions suggested by Shoman et al. were used to facilitate comparison of our results with others [13]. Dizziness was subclassified as follows: Lightheadedness, unsteadiness, vertigo, and nonspecific dizziness [13]. The onset of dizziness was also assessed and divided into three categories: early, delayed and late. Early dizziness was defined as onset of dizziness within the first post-operative week, delayed (after the first week but during the first year after implantation), and late onset (more than one year after implantation).

The data were electronically compiled and statistical analysis was performed using SPSS (20.0). Analysis of variance (ANOVA) was used to detect significant differences between the groups.

## Results

We sent 309 questionnaires. The response rate was 39% (122 patients). The mean age was 59 years and 43% of the population were males.

### Prevalence of vestibular symptoms

**Figure 1:**
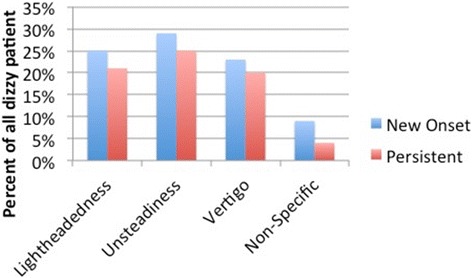
**The different subtypes of dizziness observed by the patients and the rate of presentation (in percent) in post-CI patients whether it is new onset or persistent dizziness.** Many patients had more than one description of their dizziness.

The prevalence of dizziness pre-implantation was 27% (33 patients), whereas, the prevalence of post implantation dizziness was 45.9%, (56 patients). These 56 patients were divided into two groups (groups C & D) depending on the presence or absence of dizziness prior to their implantation (Table 1). Unsteadiness was the most common subtype of dizziness (25 and 29% for groups C and D respectively) followed by lightheadedness (25 and 21% respectively). The incidence of new onset vertigo was 23% of patients complaining of dizziness (Figure 1).

**Table 1:** **Classification of the different groups of the patients depending on their dizziness symptoms**

	**Number (% of total 122)**
Group A (No dizziness)	56 (45.9%)
Group B (Dizziness only preoperative)	10 (8.2%)
Group C (persistent dizziness)	23 (18.9%)
Group D (new onset dizziness)	33 (27%)

In the patients in group C dizziness started early in most of the patients, whereas in group D the symptoms were either early or delayed. These results were not statistically significant (p >0.05).

### Dizziness handicap inventory

**Figure 2:**
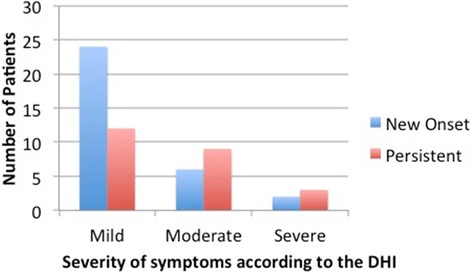
**The severity of the dizziness in patients with persistent or new onset dizziness according to the DHI score.**

In most cases, the patients reported that dizziness, whether persistent or new onset, was mild. This is illustrated in Figure 2.

The DHI score was found to be higher in the group of patients who had dizziness prior to CI, but the results were not statistically different (p = 0.096) (Table 2). On further sub-analysis of the different DHI categories, the mean DHI score for physical disability was 11 in the group that had dizziness prior and after CI compared to 7 in the group who had dizziness only after CI. These results were statistically significant (p = 0.019) (Tables 2).

**Table 2:** **The different categories of DHI and their impact on CI patients with dizziness**

**DHI**	**Persistent dizziness**	**New-onset**	**Significance (p)**
Physical	11.1	7.2	0.019
Emotional	8.4	6.3	0.338
Functional	12.5	8.5	0.139
Total	32	22	0.096

## Discussion

This study demonstrated that post-CI dizziness was mostly mild. In fact, based on the DHI scores, it can be concluded that the quality of life was relatively good and the incidence of new onset dizziness after CI was relatively low. Additionally, 27% of the patients had dizziness prior to the implantation and in almost 1/3 of these patients it resolved after the implantation.

This study was designed to parallel the methodology in Shoman et al. Interestingly, Shoman et al. reported that 48.3% of patients suffered from dizziness prior to CI (vs 27% in this study), and 58.2% post implantation (vs 45.9% in this study) [13]. Kubo et al. reviewed 94 patients, and 49% of them had post-CI dizziness [14]. This is quite similar to the prevalence in this study.

We further sought to determine when post-implantation the dizziness started. Our results show that 45.4% of our patients had delayed onset of dizziness. Whereas Shoman et al. reported onset of dizziness within one week in 63.4%. Kubo et al. also found that only 16% had delayed new onset dizziness after implantation.

Dizziness was broken down into four subcategories: unsteadiness, lightheadedness, vertigo and nonspecific. Unsteadiness was the most common symptom this is represented by 53% out of the patients who were dizzy post implantation (groups C & D). This is similar to previously reported results. Furthermore, new onset vertigo was not very common post-CI, and only less than 10% of implanted patients suffered vertigo (13 patients out of 122). This incidence was somewhat less but comparable to the findings of Kubo et al. [14]. They also mentioned that vertigo post implantation was usually delayed and prevalent in 16% patients [14].

Despite a high proportion of patients reporting dizziness related complaints, the results of this study are encouraging, since most of the patients had mild symptoms. This was reflected by a low DHI (validated and reliable questionnaire in assessing the impact of dizziness on patient’s daily life subjectively in three domains: functional, emotional and physical [11,12]. The mean DHI for the new onset dizziness group was 22 (mild) compared to 32 (moderate) for persistent dizziness group. These results were very similar to those reported by Shoman et al. [13]. The low DHI for most patients and the low incidence of reported new onset vertigo (10%) correlate with the clinical impression that most patients are relatively well from a balance perspective after implantation. This becomes an important tool in counseling the patients, who can be reassured that despite surgical opening and entering their inner ear, dizziness related should be mild.

The disappearance of dizziness in some patients after implantation is noteworthy. It might be that with improved auditory function these patients were able to use auditory clues to improve postural stability. Given the opening of the inner ear and the insertion of the array, it is not surprising that some patients experienced the onset of dizziness. More work is needed to quantify and understand the physiological changes in vestibular function (canals, otoliths) that result from implantation.

Unfortunately, there are limitations to this study. The main limitation is that this is a questionnaire based study and exposed to recall bias. Furthermore, while our response rate is comparable to other mailed questionnaire studies, there is always a possibility that those who did not respond to the questionnaire could be different than those who did. Additionally our study does not have a control group to look at the effects of surgery (independent of the actual implantation) or anesthesia on dizziness. However, it is not unreasonable to expect that the effects of the anesthesia would have resolved in the months or years after the procedure.

To our knowledge, to date, this is the largest study assessing the patients’ outcome in terms of dizziness. Further studies are necessary to augment the finding of this study. In the future, it would be interesting to analyze patients who have bilateral implantation and compare their vestibular function to the group of patients with single implantation. This study could be used for future systematic reviews and meta-analysis to further understand the patients symptomatology post CI.

## Conclusion

Dizziness is a common symptom in patients undergoing cochlear implant. It is usually not severe, and one third of those patients with pre-existing dizziness tend to improve post-implantation. Understanding the patients’ outcome after CI is important in counseling patients preoperatively and managing them postoperatively.

## Consent

Written informed consent was obtained from the patients for the publication of this report.

## References

[1] Djourno A, Eyries C, Vallancien B (1957). Electric excitation of the cochlear nerve in man by induction at a distance with the aid of micro-coil included in the fixture. C R Seances Soc Biol Fil.

[2] Krause E, Louza JP, Wechtenbruch J, Hempel JM, Rader T, Gurkov R (2009). Incidence and quality of vertigo symptoms after cochlear implantation. J Lar Otol.

[3] Buchman CA, Joy J, Hodges A, Telischi FF, Balkany TJ (2004). Vestibular effects of cochlear implantation. Laryngoscope.

[4] Bouccara D, Esteve Fraysse MJ, Loundon N, Fraysse B, Garabedian N (2005). Sterkers O [Vestibular dysfunction after cochlear implantation: a national multicenter clinical study]. Revue de laryngologie.

[5] Black FO, Lilly DJ, Peterka RJ, Fowler LP, Simmons FB (1987). Vestibulo-ocular and vestibulospinal function before and after cochlear implant surgery. Ann Otol Rhinol Laryngol.

[6] van den Broek P, Huygen PL, Mens LH, Admiraal RJ, Spies T (1993). Vestibular function in cochlear implant patients. Acta Otolaryngol.

[7] Katsiari E, Balatsouras DG, Sengas J, Riga M, Korres GS, Xenelis J (2013). Influence of cochlear implantation on the vestibular function. Eur Arch Otorhinolaryngol.

[8] Todt I, Basta D, Ernst A (2008). Does the surgical approach in cochlear implantation influence the occurrence of postoperative vertigo?. Otolaryngology.

[9] Melvin TA, Della Santina CC, Carey JP, Migliaccio AA (2009). The effects of cochlear implantation on vestibular function. Otology & neurotology.

[10] Fina M, Skinner M, Goebel JA, Piccirillo JF, Neely JG, Black O (2003). Vestibular dysfunction after cochlear implantation. Otology & neurotology.

[11] Jacobson GP, Newman CW (1990). The development of the dizziness handicap inventory. Arch Otolaryngol.

[12] Steenerson RL, Cronin GW, Gary LB (2001). Vertigo after cochlear implantation. Otol Neurotol.

[13] Shoman N, Ngo R, Archibald J, Pijl S, Chan S, Westerberg BD (2008). Prevalence of new-onset vestibular symptoms following cochlear implantation. J Otolaryngol.

[14] Kubo T, Yamamoto K, Iwaki T, Doi K, Tamura M (2001). Different forms of dizziness occurring after cochlear implant. Eur Arch Otorhinolaryngol.

